# Who lives in overcrowded households in north-east London? Cross-sectional study of linked electronic health records and Energy Performance Certificate register data.

**DOI:** 10.23889/ijpds.v7i3.1827

**Published:** 2022-08-25

**Authors:** Marta Wilk, Carol Dezateux, Silvia Liverani, Gill Harper

**Affiliations:** 1 Queen Mary University of London

## Objectives

Household overcrowding is associated with adverse health outcomes, including increased risk of infectious diseases, mental health problems, and poor educational attainment. We investigated inequalities in overcrowding in an urban, ethnically diverse, and disadvantaged London population by pseudonymously linking electronic health records (EHR) to Energy Performance Certificates (EPC) data.

## Approach

We used pseudonymised Unique Property Reference Numbers to link EHRs for 1,066,156 currently registered patients from 321,318 households in north-east London to EPC data.

We measured household occupancy and derived the bedroom standard overcrowding definition (number of rooms relative to occupants’ sex and ages) to estimate overcrowding prevalence. We examined associations with: household composition (adults only, single adult+children, ≥2 working-age adults+children, ≥1 retirement-age adults+children, three-generational household); ethnic background (White, South Asian, Black, Mixed, Other, missing); and Index of Multiple Deprivation (IMD) quintile. We used multivariable logistic regression to estimate the adjusted odds (aOR) and 95% Confidence Intervals (CI) of overcrowding.

## Results

Overall, 243,793 (22.9%) people were overcrowded. People living in households with children, or three-generational households were more likely (aOR [95% CI] 3.79 [3.74 - 3.84]; 6.53 [6.41 - 6.66] respectively), and single adults or retirement age adults with children less likely (0.36 [0.35 - 0.38]; 0.36 [0.23 - 0.57] respectively), to be overcrowded. Overcrowding was more likely among people from Asian or Black ethnic backgrounds (1.24 [1.22 - 1.25] and 1.17 [1.15 - 1.19] respectively). There was a dose-response relationship between IMD quintile and overcrowding: OR 0.20 [0.20 - 0.21] in the least deprived compared to most deprived quintile.

## Conclusion

One in five people in north-east London live in overcrowded households with marked inequalities by ethnicity, household generational composition, and deprivation. Up-to-date estimates of household overcrowding can be derived from linked housing and health records and used to evaluate the impact of economic policies on health and housing inequalities.

